# Cyclopædia of the Practice of Medicine

**Published:** 1881-04

**Authors:** 


Cyclopædia of the Practice of Medicine. Edited by Dr. Von Ziemssen, Professor of Clinical Medicine in Munich, Bavaria. Vol. IX. Diseases of the Liver and Portal Vein with the Chapter Relating to Intestinal Pneumonia. Albert H. Buck, M.D., New York, Editor American Edition. Wm. Wood & Co. 1880.

By far the majority of physicians have subscribed for this work, and we are satisfied that they have thus far had no occasion to regret the outlay required in its purchase. It has been a standard for reference and general study surpassed by no other work in the English language. We greet with much pleasure this ninth volume, which treats of diseases of the liver and portal vein and intestinal pneumonia.

---

Pamphlets Received: Drainage for Health, or Easy Lessons in Sanitary Science. By Joseph Wilson, M.D., Medical Director U. S. Navy.—Presly Blakiston, Philadelphia. 1881.

Health Primers: The Heart and its Function. D. Appleton & Co., New York. 1881.

The Digestion and Assimilation of Fat in the Human Body. An epitome of Laboratory Notes on Physiological and Chemical Experiments on this subject. By H. Crichett Bartlett, Ph. D., F.C.S. J. & A. Churchill, London. 1877.

On Quebracho Bark. Botanico Pharmacognostic Essay. By Dr. Adolph Hansen, (assistant at the Botanic Institute of Erlangin). Reprint from the *Therapeutic Gazette.* 1880.

Report of the Commissioner of Education for 1878.

First Bienniel Report of the North Carolina Board of Health, 1879–1880.

